# The predictive value of C-reactive protein to albümin ratio for ascending aort progression in patients with ascending aortic diameter of 40–50 mm

**DOI:** 10.1186/s13019-022-02003-5

**Published:** 2022-10-04

**Authors:** Ahmet Dolapoglu, Eyüp Avci, Tuncay Kiris

**Affiliations:** 1 Department of Cardiovascular Surgery, Balikesir University Medical Faculty, Balıkesir, Turkey; 2 Department of Cardiology, Balikesir University Medical School, Balıkesir, Turkey; 3 Department of Cardiology, Izmir Katip Celebi University, Ataturk Training and Research Hospital, Izmir, Turkey

**Keywords:** Inflammation, Ascending aorta, Aneurysm, C-reactive protein-to-albumin ratio, Computed tomography

## Abstract

We aimed to investigate the ability of the C-reactive protein-to-albumin ratio (CAR) to predict ascending aorta progression in patients with 40–50 mm diameter of ascending aortic dilatation. A total of 182 diagnosed patients with ascending aortic diameters of 40–50 mm were enrolled in this study. The study population was divided into tertiles based on yearly ascending aortic growth rate values. Group I (n = 137) was defined as a value in the lower 2 tertiles (ascending aorta growth ≤ 1.00 mm/year), and group II (n = 45) was defined as a value in the third tertile (ascending aorta growth > 1.00 mm/year). Hypertension, chronic obstructive pulmonary disease, positive family history, and CAR were found to be independent risk factors for ascending aorta growth > 1.00 mm/year. The area under the ROC curve (AUC) of CAR was 0.771(95% CI 0.689–0.854) for predicting ascending aorta growth > 1.00 mm/year. In patients with 40–50 mm ascending aneurysms, CAR may be useful to predict ascending aorta progression.

## Introduction

Ascending aortic aneurysm is a potentially life-threatening condition as it may lead to rupture or dissection [1]. Although there are certain risk factors such as male sex, smoking, hypertension, previous history of cardiovascular disease (CVD), familial disposition, and advanced age for abdominal aortic dilatation, these among the patients with ascending aortic aneurysms are not well described [1].

The pathology of aortic dilatation is varied based on its location. Cystic medial degeneration is the most important cause of ascending aortic dilatations. on the other hand, atherosclerosis is the most prominent factor for descending and abdominal aortic dilatations [1]. The association between abdominal aortic aneurysms and inflammation is previously studied and some markers such as white blood cell (WBC), C-reactive protein (CRP), interleukin 6, and albumin are shown to be responsible [2–5].

In the last few years, it has been proven that inflammation plays an important role in the development of ascending aorta dilatation. T lymphocytes and macrophages are common features in the aorta of patients with medial degeneration [6]. Inflammatory infiltration of T cells expressing Fas may contribute to ascending aortic dilatations in a similar fashion as to abdominal aortic dilatations [7]. It was found that patients with ascending aortic dilatation have elevated plasma d-dimers and hypersensitive CRP [8]. The CRP-to-albumin ratio (CAR) was found a better indicator of the inflammatory process than either marker alone in cardiovascular disease development and also the progression of abdominal aortic aneurysms [9–12]. The aim of the presented study was to investigate the predictive value of CAR for ascending aorta progression in patients with 40–50 mm ascending aneurysms.

## Materials and methods

This retrospective observational study included 182 diagnosed patients with ascending aortic diameters of 40–50 mm between January 2017 and December 2020. Patients required at least two chest computed tomographies (Philips Computed Tomography) at follow-up. Once the ascending aorta dilatation was diagnosed, computed tomographies (CT) follow-up was done 6 months later and 6–12 months thereafter based on aortic size. Aortic measurements were performed at the aortic root and mid-ascending aorta. Aortic root measurements were performed from a short axis view, mid-ascending aorta was measured on a plane strictly perpendicular to the main axis of the aorta, at the larger AA level. Ascending aorta was measured by semi-automatic by software from inner edge to inner edge in all patients. In all patients,a non-ionic iodinat Iv contrast agent (Ultravist 300 mg/m L, Schering, Berlin, Germany) with a density of 300 mmol was injected from antecubital vein using an automatic injector at a rate of 3 ml/sec. The density measurement of ascending aorta was performed automatically. The largest diameter of the ascending aorta was entered as maximal ascending aortic diameter and we used this for comparison throughout the study period. Maximum aortic diameters of the ascending aorta were compared throughout the study period. Aorta enlargement progression at the ascending aorta was analyzed by the annual growth rate defined as the difference between the diameter at the last control and the diameter at the first study divided by the follow-up time interval in years. Aortic replacement procedures performed during the study were identified and the reasons leading to the surgery were defined. Patients were censored if they underwent aortic surgery. The study population was divided into tertiles based on yearly ascending aortic growth rate values. Group I (n = 137) was defined as a value in the lower 2 tertiles (ascending aorta growth ≤ 1.00 mm/year), and group II (n = 45) was defined as a value in the third tertile (ascending aorta growth > 1.00 mm/year). We excluded patients with liver or biliary or renal diseases, hemolytic disorders, pancreatitis, pregnancy, secondary HT, connective tissue diseases, malignancy, chest pain, active infection, chronic inflammatory disease or use of hepatotoxic and antioxidant medications, bicuspid aortic valve, aortic coarctation or other congenital disorders, genetic syndromes, previous aortic valvuloplasty, corrective aorta surgery, aortic valve endocarditis, left ventricular dysfunction (EF < 40%), severe valvular dysfunction and ascending aorta dilation > 50 mm in the baseline study. The study was approved by the Ethics Committee of our hospital and complied with the Declaration of Helsinki.

### Blood sampling and hematological and biochemical analyses

The biochemical markers CRP and albumin, at baseline, or during hospitalization were extracted wherever available. Hematologic indices were measured by an automated hematology analyzer system. The albumin and CRP levels were measured using (Beckman Coulter Diagnostics). The CAR was calculated as the ratio of CRP to the albumin level.

### Statistical analysis

Statistical tests were performed with SPSS version 26 (SPSS Inc., Chicago, IL, USA). Continuous variables were presented as mean ± standard deviation, and categorical variables were shown as a number of subjects, with the percentage of the total number. Either the Student's t-test or the Mann–Whitney U test was used to compare parametric values between the two groups, as appropriate. The Chi-squared test was used to compare categorical variables. The Kaplan–Meier method was used to obtain event-free cumulative survival. Significant differences in the survival curves were shown by the A log-rank test. The factors entered into the multivariate model included those with *p* values < 0.1 from the univariate analysis and variables with known predictive values. A multivariate Cox regression analysis was used to identify independent predictors for ascending aorta growth rate. The predictive values of CAR and multivariable model plus CAR were estimated by the areas under the receiver operating characteristic curve. We used the DeLong test to compare the area under the curve (AUC) with each of these parameters [13]. Moreover, the increased discriminative value of these model was also estimated using net reclassification improvement (NRI) and integrated discrimination improvement (IDI) [14]. Two-sided *p* values < 0.05 were considered statistically significant.

## Results

182 patients with 40–50 mm ascending aneurysms were included in the study. The median follow-up period is 2.6 (1.9–3.5) years. The demographic characteristics of the patients included in the study are presented in Table 1. Group I was older than group II (47.6 ± 4.9 vs 45.5 ± 5.8, *p* = 0.034). Compared with group I, histories of hypertension (56% vs 16%), chronic kidney disease (19% vs 5%), chronic obstructive pulmonary disease (9% vs 2%) and familial disposition (34% vs 10%) were more frequent in group II (each *p* < 0.05).

**Table 1:** Demographic and clinical characteristics of patients

Variable	Ascending Aorta growth ≤ 1.00 mm/year	Ascending Aorta growth > 1.00 mm/year	*p* value
Age (years)	45.5 ± 5.8	47.6 ± 4.9	0.034
Female gender n (%)	54 (39)	15 (33)	0.466
DM n (%)	9 (7)	3 (7)	0.982
Hypertension n (%)	22 (16)	25 (56)	< 0.001
Peripheric arterial disease n (%)	2 (2)	2 (4)	0.236
COPD n (%)	3 (2)	4 (9)	0.043
Smoking n (%)	23 (27)	7 (22)	0.567
Previous stroke n (%)	1 (1)	2 (4)	0.090
Positive familial history n (%)	14 (10)	14 (34)	0.001
Previous PCI n (%)	7 (5)	1(2)	0.412
LVEF (%)	53.9 ± 5.0	54.0 ± 4.6	0.980
Type of aortic dilatation patterns			0.485
Aortic root dilatation n (%)	93 (68)	28 (62)	
Tubular dilatation n (%)	44 (32)	17 (38)	

*CAD* coronary artery disease, *DM* diabetes mellitus, *LVEF*left ventricular ejection fraction, *COPD *chronic obstructive pulmonary disease,* PCI* percutaneous coronary intervention

All laboratory results of the patients are presented in Table 2. The serum albumin levels was significantly lower in group II than in group I (4.1 ± 0.5 vs.4.3 ± 0.6, *p* = 0.024). The patients in group II had higher levels of C-reactive protein (CRP) than in group I (5 [3.5–6.0] vs 3.0 [2.0–4.0], *p* < 0.001, respectively). Moreover, CAR was higher in group II compared with group I (1.21 [0.89–1.50] vs 0.67 [0.51–0.86], *p* < 0.001).The independent predictors for identified using the multivariate logistic regression analysis are presented in Table 3. The CAR was independent predictor of ascending aorta growth > 1.00 mm/year (OR: 1.854, 95%CI1.023–3.359, *p* = 0.042, Table 3). In addition, hypertension, positive family history, and chronic obstructive pulmonary disease (COPD) were independent risk factors for ascending aorta growth > 1.00 mm/year in the multivariate logistic regression analysis.

**Table 2:** Laboratory findings of patients

Variable	Ascending Aorta ≤ 1.00 mm/year	Ascending Aorta > 1.00 mm/year	*p *value
BSA (m^2^)	1.9 ± 0.1	1.9 ± 0.2	0.906
Baseline ascending size (mm)	44.9 ± 3.1	45.1 ± 2.8	0.584
Albumin (mg/dl)	4.3 ± 0.6	4.1 ± 0.5	0.024
WBC (× 10^3^/μL)	6.7 ± 1.3	7.1 ± 1.4	0.033
Neutrophil count	4.3 ± 1.1	4.9 ± 1.2	0.001
Lymphocyte count	1.6 ± 0.3	1.5 ± 0.4	0.003
Hemoglobin (mg/dl)	13.6 ± 1.1	13.2 ± 1.4	0.025
Platelet (× 10^3^/μL)	187.4 ± 49.9	153.7 ± 48.7	0.291
CRP (mg/dl)	3.0 (2.0–4.0)	5 (3.5–6.0)	< 0.001
Creatinine (mg/dl)	0.90 ± 0.20	0.90 ± 0.27	0.862
Uric asid (mg/dl)	4.3 ± 1.2	5.5 ± 1.6	< 0.001
NLR	2.8 ± 1.2	3.7 ± 1.7	< 0.001
CAR*	0.67 (0.51–0.86)	1.21 (0.89–1.50)	< 0.001

*BSA* body surface area*, WBC* white blood cell, *CAR* C-reactive protein-to-albumin ratio,* CRP* C-reactive protein, *NLR* neutrophil-to-lymphocyte ratio

^*^Values are presented as medians with interquartile range in parentheses

**Table 3:** Independent predictors of ascending aorta growth > 1.00 mm/year

Variable	Univariate			Multivariate		
	OR	95% CI	*p* value	OR	95% CI	*p* value
Age	1.057	1.000–1.117	0.052			
Hypertension	4.276	2.362–7.741	< 0.001	3.346	1.795–6.237	< 0.001
Positive family history	2.202	1.167–4.155	0.015	2.077	1.036–4.164	0.039
COPD	3.663	1.295–10.365	0.014	3.822	1.320–11.067	0.013
Stroke	3.500	0.841–14.564	0.085			
Albumin levels^*^	0.574	0.348–0.947	0.030			
CRP*	1.377	1.193–1.589	< 0.001			
WBC^*^	1.000	1.000–1.000	0.040			
NLR	1.229	1.058–1.428	0.007			
Neutrophils^*^	1.001	1.000–1.005	0.006			
Lymphocytes^*^	0.999	0.998–1.000	0.016			
Hemoglobin	0.747	0.585–0.954	0.019			
Uric aside	1.530	1.256–1.862	< 0.001			
CAR	2.961	1.784–4.917	< 0.001	1.854	1.023–3.359	0.042

*OR* odds ratio, *CI* confidence interval, *WBC* white blood cell, *CRP* C-reactive protein, *COPD* chronic obstructive pulmonary disease, *NLR* neutrophil to lymphocyte ratio, *CAR* C-reactive protein to albumin ratio

^*^These parameters were not entered to the model in order to prevent multicollinearity

**Fig. 1:**
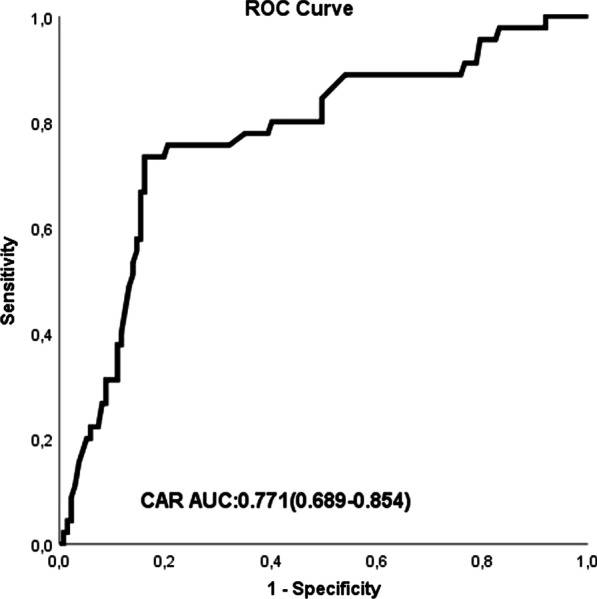
Receiver operating characteristic (ROC) curve for the CRP to albumin ratio (CAR) for predicting ascending aortic progression

**Fig. 2:**
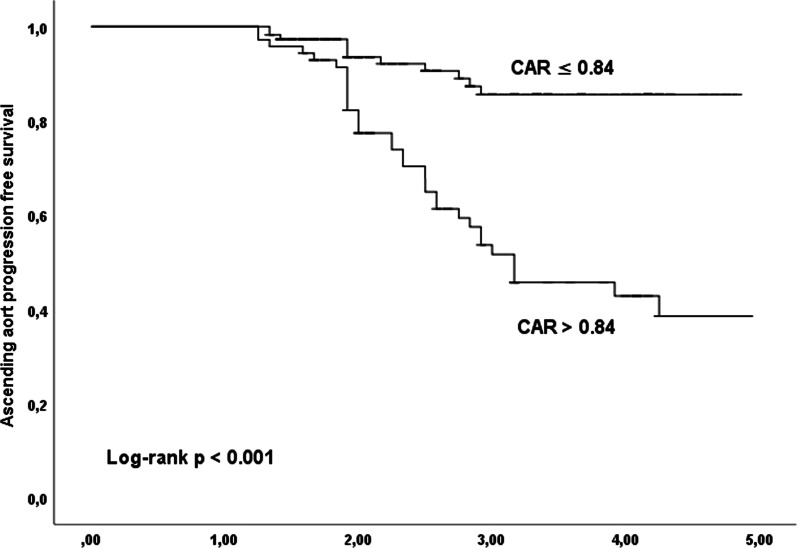
Kaplan–Meier event-free survival curves according to CAR

CAR showed a good predictive value for ascending aorta progression with an AUC of 0.771 (95% CI 0.689–0.854) (Fig. 1). Kaplan–Meier survival curve analysis according to a CAR of 0.84 as the cut-off point showed that patients with a high point had a higher cumulative incidence of ascending aorta growth > 1.00 mm/year (log-rank, *p* < 0.001) (Fig. 2).

**Fig. 3:**
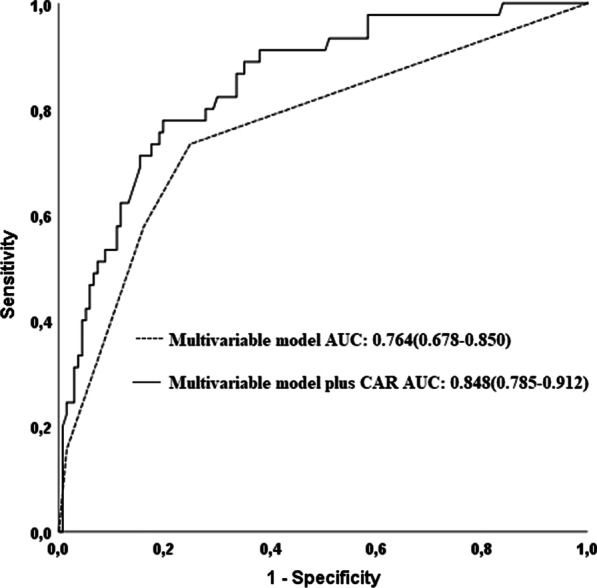
Receiver operating characteristic (ROC) curves for the multivariable model, and the multivariable model plus CAR for predicting ascending aortic progression

**Fig. 4:**
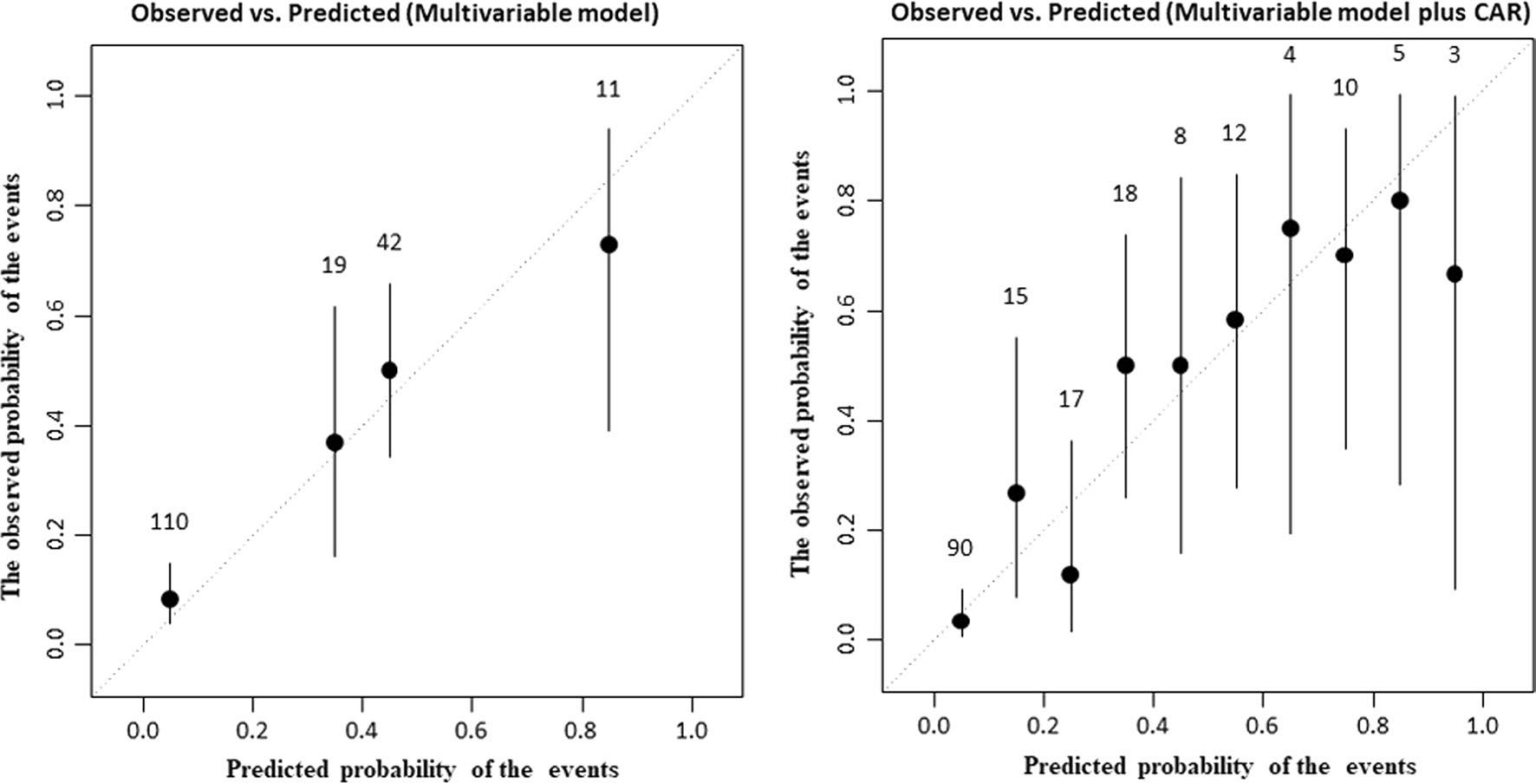
Calibration plots of the multivariable model, and the multivariable model plus CAR

Compared with multivariable model including hypertension, positive family history, and COPD, multivariable model plus CAR had higher accuracy for predicting of ascending aortic progression (multivariable model vs multivariable model plus CAR; AUC: 0.848 vs 0.764, z = 3.166, *p* = 0.0015, Fig. 3), addition of CAR to the multivariable model resulted in a NRI of 96.9% (z = 5.6132, *p* =  < 0.0001), and an IDI of 0.091 (*p* < 0.05). Calibration plots of these models were provided in Fig. 4.

## Discussion

The presented study found a clear relationship between CAR with ascending aorta dilation progression in patients with 40–50 mm ascending aortic aneurysms.

An aortic aneurysm is the result of multifactorial processes. Inflammation, genetic deformities, apoptosis, biomechanical wall stress, and proteolytic degradation of connective tissue including elastin and collagen may lead to the development of an aortic aneurysm [15, 16]. Risk factors such as hypertension increase the stress on the aortic wall, aging, inflammation, and smoking accelerate the deterioration of the fibers in the aortic wall, and some genetic factors including Marfan syndrome, Ehler-Danlos predispose the aortic wall to degeneration. A previous study published by Pasha et al. showed that increased flow-based and structural descriptors of ascending aortic aneurysms were related to high levels of circulating biomarkers including matrix metalloproteinases (MMP), tissue inhibitors of metalloproteinase (TIMP), and exosomal level of miRNA in those with ascending aortic dilatation [17]. High serum transforming growth factor-β1 (TGF-β1) to endoglin (ENG) ratio as an unfavorable TGF-β1–related gene expression profile was found in the ascending aorta in patients with the bicuspid aortic valve and is associated with a faster growth rate of the aorta over time, a surrogate marker in predicting the severity of the aortopathy [18]. It has been shown that aortic size and degree of valve morphology differentially modulate microRNA(miRNA) analytes and protein proteolytic activity in patients with ascending aortic dilatation [19]. All of them are associated with the development of aortic dilatation [15–19].

Pathologic samples of aortic tissue in patients with Marfan patients, familial thoracic aortic aneurysm (TAA), and sporadic TAA contain increased macrophage infiltration [20, 21]. IL-6 is also an inflammatory cytokine that plays an important role in aortic aneurysms [22]. Knockout of IL-6 in mouse models decreases in TAA formation and aortic size [23]. In addition, TAA tissue from human patients exhibits increased IL-6 levels [24].

C-reactive protein is a positive acute-phase reactant that may debilitate endothelial progenitor cells and increases collagen deterioration and platelet activation in the chronic inflammation process [25]. A former study has shown that CRP lead to arterial damage thus contributing to the development of aortic dilatation [26]. Albumin as a negative acute-phase protein has many biological functions such as antioxidant and anticoagulant activity, forming of vascular integrity, causation of vasodilating effects, and binding activity of drug, toxin, and cholesterol transport activation [27, 28]. It is known that reduction in albumin levels is associated with the development of inflammatory conditions or malnutrition [29, 30]. Changes in synthesis and catabolism of albumin may be related to increased inflammation and may be an important confounding factor for the prognostic utility of serum albumin [31, 32]. In addition, the formation of anti-inflammatory mediators such as lipoxins, resolvins, and protectins may be included by albümin metabolism [33]. High CRP and low albumin levels were seen in patients with ascending Aorta > 1.26 mm/year in the presented study.

It has been shown that CAR may be a marker of the balance between CRP and albumin in the body and also reflect the inflammatory and nutritional status of a patient’s condition [34]. The combination of albumin and CRP in a single index as CAR was found to be more strong with prognosis than either CRP or albumin alone [35–37]. In a previous study, it has been shown that serum CAR was related to the presence and progression of abdominal aortic aneurysms [11]. Higher white blood cell count (WBC) and neutrophil to lymphocyte ratio (NLR) as markers of inflammation were found to be associated with abdominal aortic aneurysms [38, 39]. In presented study, group I had elevated WBC count and NLR compared with group II. There was a relationship of COPD and HT to ascending aortic dilatation in previous studies, [40, 41] as found in our study.

Our study has some limitations. This study was a single-center observational study. Although follow-up was conducted, further review was not carried out. Therefore, a prospective, large-scale, and multi-center study is needed to confirm these conclusions. This study included only admission CAR, future research is to consider calculation CAR with dynamic monitoring, to evaluate CAR affecting ascending aortic aneurysm patient outcome at different time points.

## Conclusions

Aortic pathology in ascending aortic aneurysms is associated with a strong inflammatory substrate. The measurement of inflammatory parameters such as CAR may be used as a marker of rapid growth in patients with ascending aortic aneurysms. Pharmaceutical agents that target inflammatory pathways may help attenuate ascending aortic aneurysm progression and prevent their complications.

## Data Availability

The datasets used and analyzed during the current study are available from the corresponding author on reasonable request.
